# Effects of Water Temperature on Growth, Hematological Measurements and Stress-Related Gene Expression of Atlantic Salmon (*Salmo salar*) Parr Reared in a Recirculating Aquaculture System

**DOI:** 10.3390/ani15203048

**Published:** 2025-10-20

**Authors:** Yujin Lee, Kyuseok Cho, Haham Kim, Hyuncheol Jeon, Seunghyung Lee

**Affiliations:** 1 Inland Fisheries Industrial Institute of ChungCheongBuk-Do, Chungju 27432, Republic of Korea; poiuy1875@korea.kr (Y.L.); kscho6146@korea.kr (K.C.); 2 Major of Aquaculture and Applied Life Sciences, Division of Fisheries Life Sciences, Pukyong National University, Busan 48513, Republic of Korea; haham7@naver.com (H.K.); conjp@naver.com (H.J.); 3 Feeds and Foods Nutrition Research Center, Pukyong National University, Busan 48547, Republic of Korea

**Keywords:** salmonids, physiological trade-off, stress makers, sustainable aquaculture

## Abstract

Water temperature is a key factor affecting the growth, feeding performance and physiological status of Atlantic salmon parr in aquaculture. To determine optimal conditions, parr (average weight 31.27 ± 0.35 g) were reared for 60 days at 10, 14, 18, and 22 °C. The survival and condition factors were similar across treatments. The growth rate and feed efficiency were highest at 14 °C, coinciding with elevated antioxidant activity. Feed intake was lowest at 10 °C. Whole-body protein and lipid contents remained unaffected, while moisture and ash contents were lowest at 14 °C. Most plasma biochemical indicators were stable; however, total protein was lowest at 14 °C. Glutathione peroxidase activity peaked at 14 °C, whereas cortisol levels remained unchanged. Heat shock proteins (HSP70, HSP90) increased with temperature, while insulin-like growth factor binding proteins (IGFBP1A, IGFBP1B) decreased at temperatures equal to or greater than 18 °C. Interferon alpha (IFNA) and thioredoxin (TRX) were lowest at 14 °C and highest at 22 °C. Overall, 14 °C appears optimal for growth and antioxidant capacity, although molecular stress markers suggest mild physiological trade-offs. These findings can inform temperature management strategies to enhance productivity and welfare in sustainable salmon aquaculture.

## Simple Summary

Atlantic salmon farming is influenced by water temperature, which can affect growth, feeding behavior, and overall health. In this study, juvenile salmon were raised for two months, at temperatures of 10, 14, 18, and 22 °C to assess their performance under different conditions. The growth was fastest at 14 °C, accompanied by efficient feed utilization and strong antioxidant activity. Most health indicators remained stable; however, some stress and immune responses varied at higher temperatures. These results suggest that 14 °C is close to optimal for growth and overall balance, though this temperature may still induce mild stress. Understanding these effects can help farmers select water conditions that enhance both productivity and fish welfare in sustainable aquaculture.

## 1. Introduction

Atlantic salmon (*Salmo salar*) is one of the most commercially valuable aquaculture species worldwide, with Norway and Chile together accounting for approximately 80% of total production [1]. In South Korea, more than 90% of imported salmon is farmed Atlantic salmon [2]. Annual imports increased from 9400 tons in 2010 to 42,000 tons in 2020, reaching a peak of 63,000 tons in 2021 and 77,000 tons in 2022. In 2023, imports decreased to 44,000 tons; however, the total import value remained comparable to that in 2022 (USD 550 million vs. USD 560 million), mainly due to rising prices driven by exchange rate fluctuations and transportation costs [3]. This heavy reliance on imports makes the domestic market vulnerable to fluctuations in international supply and demand, highlighting the urgent need to develop local production capacity [4].

Until recently, Atlantic salmon cultivation in Korea was restricted, as the species was classified as ecologically hazardous in 2016 [5]. However, the 2021 amendment to the Ministry of Environment’s regulations (Notification No. 2021-45) authorized the importation of fertilized eggs for the first time, enabling pilot-scale aquaculture trials. Since then, national and local governments have implemented policies and research initiatives aimed at stabilizing supply and prices through the development of domestic Atlantic salmon farming. To meet increasing demand and stabilize fish supply, it is imperative to advance aquaculture technologies and practices tailored specifically to Korea’s aquatic environments.

Among the various aquatic environmental factors, water temperature is a critical determinant for fish, especially ectothermic species whose body temperature is directly influenced by ambient conditions [6]. It regulates key physiological processes, including metabolic rate, feeding behavior, feed intake, digestion rate, and digestive enzyme activity. Optimal thermal ranges promote maximum growth, whereas deviations can lead to growth reduction [7,8,9]. Temperature also plays a vital role in immune function, with both innate and adaptive immunity functioning effectively within an optimal temperature range. Conversely, extreme high or low temperatures can impair immune responses and increase susceptibility to disease [10].

In salmonids, water temperature additionally influences embryonic development, sexual maturation, and smoltification [11,12,13,14]. Temperatures outside the optimal range can induce physiological stress, elevating cortisol secretion and diverting metabolic energy toward recovery and homeostasis. Under such conditions, antioxidant and immune-related gene expression often increase, accompanied by enhanced enzyme activity, immune protein synthesis, and antibody production [15]. Recently, RNA-seq-based transcriptomic analyses have identified thermal stress pathways in salmonids, including heat shock proteins (HSPs), oxidative stress, and immune-related genes, thereby providing molecular insights into the responses to temperature fluctuations [16].

The optimal growth temperature for Atlantic salmon parr in natural environments has been reported to be 18–19 °C, while the optimal feeding temperature is 19.5–19.8 °C [17]. These thermal windows are not only critical for understanding physiological responses during the parr stage but also provide practical guidance for aquaculture management. In particular, triploid salmon are widely used in commercial farming to suppress early maturation, and they have been shown to differ from diploids in thermal tolerance and metabolic responses. Therefore, their specific physiological traits should also be considered when evaluating temperature effects [18,19,20].

Determining the optimal rearing temperature for each life stage is therefore essential to minimize stress, promote healthy growth, and ensure sustainable production. Fish health assessments typically incorporate morphometric growth measurements, hematological and biochemical analyses, and the evaluation of biomarker genes that respond specifically to environmental stressors [21,22,23]. Advances in genomic research on aquatic organisms have further enhanced the utility of such biomarkers in evaluating environmental adaptation and health status [23]. Recently, studies from Korea have also reported the significant influence of water temperature on the growth and stress-related gene expression of salmonids, emphasizing the need for region-specific investigations [24,25]. Against this background, the present study aims to determine the optimal rearing temperature for domestically farmed Atlantic salmon by evaluating survival, growth performance, hematological and biochemical parameters, and the expression of selected biomarker genes under different water temperature conditions.

## 2. Materials and Methods

### 2.1. Experimental Fish and Rearing Conditions

The current feeding trial was reviewed and approved by the Institutional Animal Care and Use Committee (IACUC) of Pukyong National University, Busan, Republic of Korea (protocol number: PKNUIACUC-2024-25).

Atlantic salmon (*Salmo salar*) were obtained as fertilized eggs (all-female triploid) imported from Benchmark Genetics, Iceland, by the Inland Fisheries Industrial Institute of ChungCheongBuk-Do. The fish were acclimated for two weeks prior to the experiment in a recirculating aquaculture system (RAS) comprising a rearing tank (1.2 × 0.8 × 0.3 m), a bio-sand filter unit (0.7 × 0.5 × 0.2 m) filled with ceramic rings and quartz sand media (particle size: 0.5~1.0 mm; total volume 50 L), and a chiller (HAK-1000, Hi Air Airconditioning, Gimhae, Republic of Korea). The trial was conducted using four recirculating rearing tanks, each divided into three independent compartments serving as replicates. Thus, a total of 12 experimental units (four temperature treatments × three replicates) were established, with 24 fish stocked per compartment.

During the acclimation period, the water quality parameters were maintained at 10 ± 1 °C, pH 7.7–7.8, dissolved oxygen 8 ± 1 mg/L, ammonia 0 mg/L, nitrite < 0.2 mg/L, and nitrate < 3 mg/L. Water temperature and dissolved oxygen (DO) were measured using a DO meter (HQ40d, YSI Inc., Yellow Springs, OH, USA), while pH was monitored with a pH meter (Pro1020, YSI Inc., Yellow Springs, OH, USA). Ammonia (NH_4_^+^-N; Cat. No. 06022-01, Humas, Daejeon, Republic of Korea), nitrite (NO_2_^−^-N; Cat. No. 04012 -01, Humas), and nitrate (NO_3_^−^-N; Cat. No. 05030-01, Humas) were measured using a multiparameter water quality meter (HS 1000 Plus, Humas), and suspended solids (SS) were analyzed using the instrument (DOA-V717-AA, GAST, Benton Harbor, MI, USA) following the manufacturer’s instructions.

The experiment was initiated at 10 °C, and the water temperature was gradually increased to the target levels of 14, 18, and 22 °C at a rate of 1 °C every 2 h using a chiller or an electric heater. Once the target temperature was reached, it was maintained within ±0.5 °C. Water temperature in each tank was monitored every minute with a data logger (Maxmin-24 V2, Elitech, San Jose, CA, USA) to ensure stable conditions. The photoperiod was set to 24 h light–0 h dark (24L:0D). Dissolved oxygen was maintained near saturation through continuous aeration.

Fish were randomly allocated to experimental tanks to minimize tank effects, and sampling was performed in a blinded manner to avoid observer bias.

### 2.2. Experimental Diet and Feeding

Fish were fed a commercial salmon diet formulated to contain 49.08% crude protein and 14.56% crude lipid. The diet composition and proximate analysis are shown in Table 1. Feed was provided to apparent satiation twice daily (09:00 and 17:00) for 60 days. Satiety was determined by observing feeding behavior until the cessation of feeding response, and uneaten feed was collected within 10 min after feeding, dried at 60 °C for 24 h, and weighed to accurately estimate feed intake.

### 2.3. Growth Performance and Sampling

At the beginning and end of the trial, fish were anesthetized with 100 mg L^−1^ tricaine methanesulfonate (MS-222, Sigma, St. Louis, MO, USA) buffered with sodium bicarbonate, and total length and body weight were recorded. This concentration is within the recommended range for surgical anesthesia in fish [27] and has also been previously applied in Atlantic salmon (*Salmo salar*) [28]. Growth parameters, including weight gain (WG), specific growth rate (SGR), feed efficiency (FE), and daily feed intake (DFI), were calculated according to standard formulas.

Survival rate (SR, %) = (final number of fish/initial number of fish) × 100.
Weight gain (WG, %) = (final weight (g) − initial weight (g))/initial weight (g) × 100.
Specific growth rate (SGR, %/day) = (ln final weight (g) − ln initial weight (g))/days × 100.
Daily feed intake (DFI, %) = feed intake/[(initial fish weight (g) + final fish weight (g) + dead fish weight (g)) × days reared/2] × 100.
Feed efficiency (FE, %) = (final weight (g) − initial weight (g))/feed consumed (g) × 100.
Protein efficiency ratio (PER) = (final weight (g) − initial weight (g))/protein intake (g).
Condition factor (CF) = (final weight (g)/total length (cm)^3^) × 100.
Hepatosomatic index (HSI, %) = liver weight (g)/body weight (g) × 100.
Viscerosomatic index (VSI, %) = viscera weight (g)/body weight (g) × 100.

### 2.4. Whole-Body Proximate Composition

Three fish per tank were randomly selected, euthanized, and homogenized for whole-body analysis. Moisture content was determined by oven drying at 105 °C for 24 h; crude protein by the Kjeldahl method (N × 6.25); crude lipid by Soxhlet extraction [29] and crude ash by combustion at 550 °C for 6 h, following the standard procedures of AOAC (2005) [30].

### 2.5. Plasma Biochemical Analysis

Plasma biochemical parameters were analyzed using a pooled sample from three fish per tank (*n* = 3 per temperature group). Blood samples were collected from the caudal vein using heparinized syringes and centrifuged at 3000× *g* for 10 min at 4 °C to obtain plasma. Plasma aspartate aminotransferase (AST), alanine aminotransferase (ALT), glucose (GLU), triglycerides (TG), total cholesterol (TCHO), and total protein (TP) were measured using FUJI DRI-CHEM slides (AST, Cat. No. 6330-AST; ALT, Cat. No. 6330-ALT; GLU, Cat. No. 6330-GLU; TG, Cat. No. 6330-TRIG; TCHO, Cat. No. 6330-CHOL; TP, Cat. No. 6330-TP; FUJIFILM, Tokyo, Japan) with an automated biochemical analyzer (DRI-CHEM 4000i, FUJIFILM, Tokyo, Japan). Plasma electrolytes (Na^+^, K^+^, Cl^−^) were determined using FUJI DRI-CHEM LYTES-P slides (Cat. No. 6330-LYTES; FUJIFILM, Tokyo, Japan). Osmolality was measured using an osmometer (Vapro 5600, WESCOR Inc., South Logan, UT, USA).

### 2.6. Antioxidant Enzyme Activity and Cortisol

GPx activity and plasma cortisol were analyzed using a pooled sample from three fish per tank (*n* = 3 per temperature group). All blood samples were collected between 13:00 and 14:00 under continuous light conditions (24L:0D) to minimize diel variation.

Glutathione peroxidase (GPx) activity was determined using a commercial kit (CSB-E15930Fh; Cusabio, Houston, TX, USA) following the manufacturer’s protocol. Plasma cortisol levels were measured using an enzyme-linked immunosorbent assay (ELISA) kit (CSB-E08487f; Cusabio, Houston, TX, USA). Absorbance was read at 450 nm within 5 min using a microplate reader. The mean intra- and inter-assay CV values were 6.3% and 7.7%, respectively. The best-fit model, selected from the curve fit software (CurveExpert 1.4, Hyams Development), was the Weibull model, with an R^2^ of 0.9969. The original sample was diluted tenfold (×10) for the analysis.

### 2.7. Gene Expression Analysis

Liver samples were collected from three fish per tank, immediately frozen in liquid nitrogen, and stored at −80 °C until RNA extraction. Total RNA was extracted using TRIzol reagent (Invitrogen, Carlsbad, CA, USA), treated with DNase I (Thermo Fisher Scientific, Waltham, MA, USA), and quality was confirmed spectrophotometrically (A260/280 = 1.9–2.1) and by RNA integrity number (RIN ≥ 7.0). Complementary DNA (cDNA) was synthesized from 1 µg of total RNA using the PrimeScript RT kit (Takara Bio Inc., Kusatsu, Japan).

Quantitative real-time PCR (qPCR) was conducted with SYBR Green Master Mix (Bio-Rad, Hercules, CA, USA) on a CFX96 system (Bio-Rad, Hercules) using the primers listed in Table 2. Each qPCR assay was run in triplicate and included no-template controls and melt-curve analysis to confirm specificity. Five-point 10-fold dilution series were used to generate standard curves. Primer efficiencies ranged from 89.7% to 104.3%, with coefficients of determination (R^2^) between 0.987 and 0.999, and Pearson’s coefficients between 0.978 and 0.998 (Table 2).

To validate reference gene stability, seven commonly used housekeeping genes in salmonids (*ef1α*, *rpl13a*, *gapdh*, *ubq*, *tuba1*, *18S rRNA*, and *β-actin*) were evaluated across all treatments and time points. Cq values were analyzed using geNorm, NormFinder, and BestKeeper algorithms. The geNorm pairwise variation analysis indicated V_2_/_3_ < 0.15, suggesting that expression stability among the candidate genes was sufficient for reliable normalization. Based on concordant results across all three algorithms, *β-actin* was identified as the most stable gene and was used as the single reference gene in this study.

Relative transcript levels of the target genes—heat shock protein 70 (*hsp70*), heat shock protein 90β (*hsp90β*), insulin-like growth factor binding protein 1A (*igfbp1a*), insulin-like growth factor binding protein 1B (*igfbp1b*), interferon alpha (*ifna1*), and thioredoxin (*trx*)—were quantified using the efficiency-corrected ΔΔ*Cq* method [31]:

(1) $$RQ\;=\frac{{\left(E_{target}\right)}^{-{\Delta Cq}_{target}}}{{\left(E_{RG}\right)}^{-{\Delta Cq}_{RG}}},\;\;\;E\;=\;10^{-1/slope}$$

where Δ*Cq* denotes (sample–calibrator) and *E* represents amplification efficiency from the standard curve. Expression data were log_2_-transformed before statistical analysis. Results are expressed as mean ± SEM, and differences were analyzed with Treatment and Time as fixed factors and Tank as a random factor.

### 2.8. Statistical Analysis

All experimental analyses were conducted in triplicate. Statistical analyses were performed using SPSS Statistics software (Version 20; IBM, Armonk, NY, USA). One-way analysis of variance (ANOVA) was used to test for differences among groups, and Tukey’s multiple range test was applied for post hoc comparisons. Differences were considered statistically significant at *p* < 0.05.

## 3. Results

### 3.1. Water Quality

During the experimental period, water temperature was maintained at 10.31 ± 0.17 °C, 14.38 ± 0.11 °C, 18.31 ± 0.08 °C, and 22.23 ± 0.09 °C for the respective treatment groups, with significant differences observed among treatments (*p* < 0.05). Dissolved oxygen (DO) levels ranged from 8.31 to 8.50 mg/L across all treatments, and pH values ranged from 7.76 to 7.86, with no significant differences detected (*p* > 0.05). Ammonia concentrations remained at 0.00 mg/L in all treatments. Nitrite concentrations ranged from 0.16 to 0.24 mg/L, and nitrate concentrations ranged from 2.69 to 3.16 mg/L, with no statistically significant differences among treatments (*p* > 0.05). Suspended solids (SS) concentrations ranged from 1.32 to 1.77 mg/L, also showing no significant differences (*p* > 0.05) (Table 3).

### 3.2. Growth Performance

The survival rate and condition factor (CF) showed no significant differences among groups (*p* > 0.05) (Table 4). At day 60, the weight gain (WG) and specific growth rate (SGR) were significantly higher in the 14 °C group, followed by the 18 °C, 10 °C, and 22 °C groups (*p* < 0.05). Daily feed intake (DFI) was significantly lower at 10 °C compared to the other groups (*p* < 0.05). Feed efficiency (FE) did not differ significantly between the 10 °C and 14 °C groups (*p* > 0.05) but decreased significantly with increasing temperature above 14 °C (*p* < 0.05). The protein efficiency ratio (PER) showed no significant difference between 10 °C and 14 °C but was lower at 18 °C and 22 °C (*p* < 0.05). The hepatosomatic index (HSI) did not differ significantly among treatments (*p* > 0.05), whereas the viscerosomatic index (VSI) was highest at 10 °C and lowest at 22 °C (*p* < 0.05).

### 3.3. Whole-Body Proximate Composition

Whole-body moisture content ranged from 69.00 to 70.19% and was significantly lower at 14 °C compared to 22 °C (*p* < 0.05), with intermediate values observed at 10 °C and 18 °C. Crude protein content ranged from 17.62 to 18.81% and did not differ significantly among treatments (*p* > 0.05). Crude lipid content ranged from 8.65 to 10.07% and also showed no significant difference (*p* > 0.05). Crude ash content was lowest at 14 °C and highest at 18 °C (*p* < 0.05), with 10 °C and 22 °C showing intermediate values (Table 5).

### 3.4. Plasma Biochemical Parameters

Plasma biochemical indices indicated no significant differences in AST, ALT, GLU, TG, TCHO, Na^+^, K^+^, Cl^−^, or osmolality among the temperature groups (*p* > 0.05). Total protein (TP) was significantly lower at 14 °C than at 22 °C (*p* < 0.05), with intermediate values observed at 10°C and 18 °C (Table 6).

### 3.5. Antioxidant Enzyme Activity and Cortisol

Plasma glutathione peroxidase (GPx) activity was highest in the 14 °C group (*p* < 0.05). Cortisol concentrations did not differ significantly among treatments (*p* > 0.05) (Table 7).

### 3.6. Gene Expression

**Figure 1:**
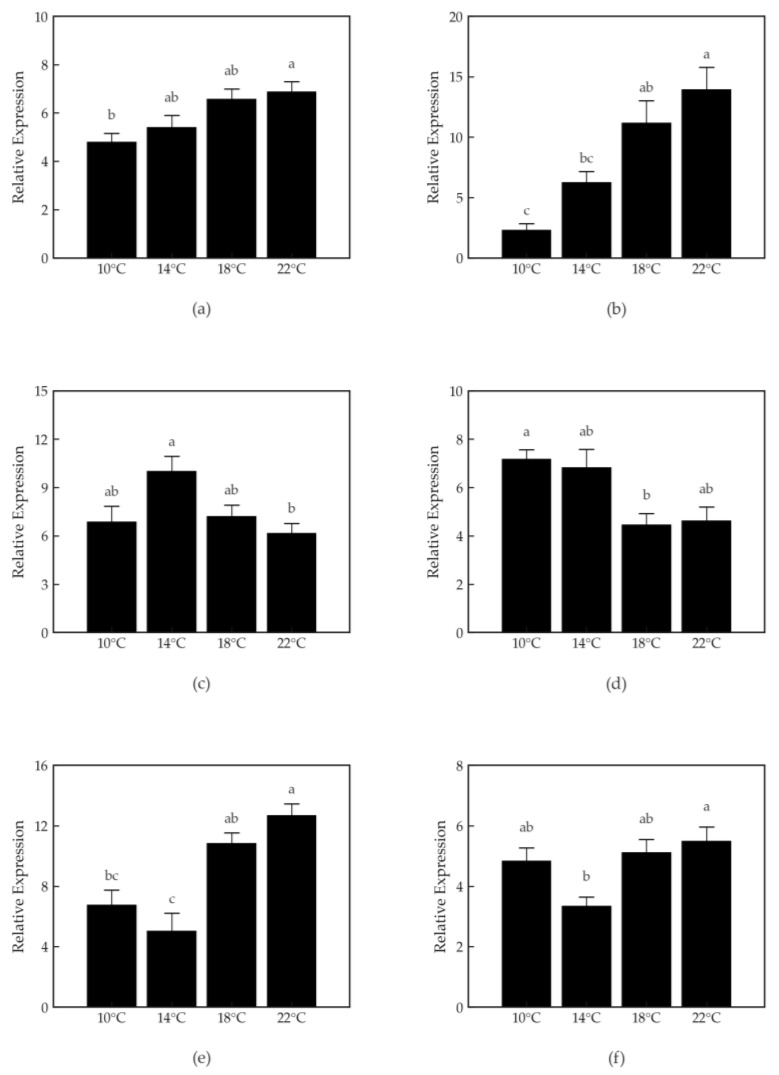
Relative expression levels of genes in Atlantic salmon parr liver tissues at different water temperatures (10 °C, 14 °C, 18 °C, and 22 °C) were measured. Data are presented as mean ± SEM (n = 3). Different lower-case letters above the bars indicate significant differences among temperatures within the same gene (one-way ANOVA followed by Tukey’s HSD test, *p* < 0.05). (**a**) *hsp70* (stress-related); (**b**) *hsp90β* (stress-related); (**c**) *igfbp1a* (growth-related); (**d**) *igfbp1b* (growth-related); (**e**) *ifna1* (immune-related); (**f**) *trx* (oxidative stress-related).

The relative mRNA expression levels of *hsp70* and *hsp90β* increased significantly with rising water temperature (*p* < 0.05) (Figure 1). The expression of *igfbp1a* and *igfbp1b* decreased significantly in the 18 °C and 22 °C groups compared to a lower temperatures (*p* < 0.05). *ifna* and *trx* expression was lowest at 14 °C and highest at 22 °C (*p* < 0.05).

## 4. Discussion

The diet used in this study was a custom-made feed formulated at the Feed and Nutrition Laboratory of Pukyong National University, and was previously demonstrated to yield high growth performance in Atlantic salmon parr. It contained 49.08% crude protein and 14.56% crude lipid, which is consistent with the recommended nutrient range for the parr stage of Atlantic salmon (≥40% protein and ≤30% lipid) [32]. An appropriate balance of protein and lipid is essential for growth, body composition, metabolic function, and immune health. This baseline supports the interpretation of growth patterns observed in this study.

Water temperature is a major environmental factor influencing fish metabolism, feeding behavior, digestion, and nutrient absorption [7,33]. Optimal growth temperatures vary among salmonid species and life stages. For example, lake trout (*Salvelinus na-maycush*) grow best at ~12.5 °C, brown trout (*Salmo trutta*) at 16 °C, Chinook salmon (*On-corhynchus tshawytscha*) at 15 °C, and coho salmon (*Oncorhynchus kisutch*) at 12–15 °C [34,35,36]. For Atlantic salmon, previous reports indicate ~10 °C during egg incubation, 16–20 °C during the first-feeding fry stage, ~18–19 °C for the parr stage, and ~13 °C for the marine grow-out stage [17,37]. However, these ranges can vary depending on experimental conditions, water quality, diet composition, and fish size.

In the present study, survival rates exceeded 95% across all temperature treatments (10, 14, 18, and 22 °C), with no significant differences observed. This aligns with the high thermal tolerance of Atlantic salmon, as the reported critical survival threshold for parr and smolt is ~30–33 °C [38]. Thus, the highest experimental temperature (22 °C) did not approach lethal limits for this life stage.

Growth performance was strongly influenced by temperature. Weight gain (WG) and specific growth rate (SGR) were highest at 14 °C, followed by 18 °C, 10 °C, and 22 °C. Feed efficiency (FE) and protein efficiency ratio (PER) were significantly reduced at temperatures above 14 °C. Such growth patterns may partly reflect the unique metabolic characteristics of triploid salmon, which often exhibit higher metabolic rates at lower temperatures and reduced efficiency under warm-water conditions [18,19,20]. At higher temperatures, the increase in basal metabolic rate and stomach evacuation rate (SER) likely shortens gut passage time and decreases nutrient absorption [39,40]. At 22 °C, while daily feed intake (DFI) increased, but this did not translate into proportional growth, leading to reduced conversion efficiency. These trends are consistent with previous studies showing impaired growth efficiency under warm-water conditions [40].

Whole-body composition analysis showed that crude lipid content and viscerosomatic index (VSI) were higher at 10 °C, likely reflecting lipid accumulation associated with lower metabolic rates [25,41]. In contrast, moisture and crude ash contents were lowest at 14 °C, possibly due to a higher proportion of protein and lipid in the body composition at this temperature.

Plasma biochemical analysis revealed no significant temperature effects on AST, ALT, glucose, total cholesterol (TCHO), or triglycerides (TG). However, total protein (TP) was highest at 22 °C and lowest at 14 °C. The higher TP concentration at 22 °C may indicate increased protein synthesis or elevated immune-related protein production in response to thermal stress [42]. The lower TP at 14 °C suggests efficient utilization of protein for growth. Plasma ion concentrations (Na^+^, K^+^, Cl^−^) and osmolality showed no significant differences among treatments, suggesting no major temperature effects on osmoregulatory balance within the tested range [43].

Cortisol, a primary stress indicator, showed no significant temperature-related differences, possibly due to thermal acclimation over the 60-day experimental period [44]. Similar findings have been reported in Atlantic salmon and rainbow trout exposed to different thermal regimes for prolonged periods, where cortisol levels initially increased but later stabilized as fish acclimated to the temperature [42,44]. This suggests that the absence of a temperature effect in the present study may reflect thermal acclimation to the rearing conditions rather than a lack of stress response [45].

Antioxidant defense, measured as glutathione peroxidase (GPx) activity, remained high at 14 °C with the GPx value (679.33 ± 90.58 mU ml^−1^) being similar to the initial level (652.90 ± 134.96 mU ml^−1^), whereas GPx activity tended to decrease at other temperatures. GPx functions as a key enzymatic antioxidant that protects cells from oxidative damage caused by reactive oxygen species (ROS) [46,47]. The stable GPx activity at 14 °C indicates that antioxidant capacity was effectively maintained under optimal growth conditions, whereas its reduction at ≥18 °C may reflect the accumulation of ROS and the onset of oxidative stress [48]. Chronic oxidative stress has been shown to induce lipid peroxidation and impair cellular integrity, ultimately compromising physiological performance [49]. This indicates that antioxidant capacity was stable at 14 °C, while deviations from this temperature may be associated with increased accumulation of reactive oxygen species (ROS) [48].

Gene expression analysis showed increased HSP70 and HSP90 expression with rising temperature, consistent with typical heat stress responses. HSPs play essential roles in protein folding, stabilization, and repair during environmental stress [50]. The upregulation of HSP70 and HSP90 at higher temperatures observed in this study is consistent with previous findings [16], which reported a strong induction of HSPs in Atlantic salmon maintained at 20 °C. Such persistent activation of cellular stress mechanisms may, however, indicate ongoing thermal stress that can negatively affect immune function and growth.

IGFBP1A and IGFBP1B expression showed the highest levels at 14 °C and 10 °C, respectively, and tended to decrease at higher temperatures, suggesting suppression of IGF signaling and potential growth inhibition under thermal stress [51]. These binding proteins regulate the bioavailability of insulin-like growth factors and are highly sensitive to temperature-driven metabolic shifts [52]. Similar trends have been reported in previous studies [51], which observed that prolonged exposure to elevated temperature (19 °C) reduced IGFBP1 expression in Atlantic salmon.

IFNA and thioredoxin (TRX) expression peaked at 22 °C, indicating activation of immune and redox homeostasis pathways under high-temperature stress [16]. The upregulation of IFNA is consistent with enhanced innate immune activation under environmental stress [16,53], while increased TRX expression suggests an attempt to restore intracellular redox balance [54]. Such responses represent compensatory mechanisms for maintaining homeostasis, but sustained activation may lead to immune exhaustion or tissue damage under chronic stress [55].

However, the experimental period of 60 days represents a short-term trial compared with commercial grow-out cycles, which typically extend over several months until smoltification and transfer to seawater [56]. As such, the present findings primarily reflect early responses during the parr stage, and the potential long-term carry-over effects on smoltification success, seawater growth performance, and sexual maturation remain to be clarified in future studies. From an industry perspective, economic feasibility must also be considered, and large-scale production trials in land-based closed RAS have already demonstrated the potential for commercial application [57].

## 5. Conclusions

This study demonstrated that rearing Atlantic salmon parr under domestic RAS conditions for 60 days resulted in optimal growth performance and antioxidant defenses at 14 °C, while higher temperatures (≥18 °C) were associated with reduced growth, increased oxidative damage, and stronger stress responses. Although HSP upregulation was observed at 14 °C compared with 10 °C, this likely reflects subtle physiological adjustments rather than measurable performance costs. Nonetheless, the short duration of the trial and the lack of long-term assessments underscore the need for extended studies. From an industry perspective, maintaining water temperature below approximately 18 °C appears advisable as a practical guideline for RAS rearing of Atlantic salmon parr.

## Figures and Tables

**Table 1:** Ingredients and proximate composition (%) of the experimental diets used in the feeding trial with Atlantic salmon (*Salmo salar*).

Ingredients	Value (%)
Fish meal	50.00
Black soldier fly meal	5.00
Soybean meal	5.00
Squid liver powder	4.10
Tankage meal	2.50
Wheat flour	21.80
Fish oil	7.80
Monocalcium phosphate	1.00
Mineral mix	1.00
Vitamin mix	1.00
Vitamin C	0.20
Choline	0.60
Total	100
Proximate analysis (% of dry matter basis)	
Moisture	2.06
Crude Protein	49.08
Crude Lipid	14.56
Crude Ash	12.31
Gross energy (MJ/kg) ^1^	23.74
Non-protein energy (MJ/kg) ^1^	11.95

^1^ The energy values of protein, lipid, and carbohydrate were calculated as 23.6, 17.2, and 39.5 kJ/g, respectively [26].

**Table 2:** Primers used for quantitative real-time polymerase chain reaction (qPCR) analysis of gene expression.

Genes	Primer Sequences	Product Size (bp)	Accession Number	Primer Efficiency	R^2^	Pearson’s Coefficient
*hsp70* ^1^	F:CCCCTGTCCCTGGGTATTG R:CACCAGGCTGGTTGTCTGAGT	121	BG933934	96.1	0.997	0.995
*hsp90β* ^2^	F:CCACCATGGGCTACATGATG R:CCTTCACCGCCTTGTCATTC	114	NM_001123532	93.4	0.994	0.989
*igfbp1a* ^3^	F:GGTCCCTGTCATGTGGAGTT R:TTCCAGAAGGACACACACCA	184	KC122927	101.8	0.998	0.997
*igfbp1b* ^4^	F:GAGGACCAGGGACAAGAGAAAGT R:GCACCCTCATTTTTGGTGTCA	101	AY662657	89.7	0.987	0.978
*ifna1* ^5^	F:CACAGGCATGGGAGCTCATC R:TGACAGGGTCCCACGTGATT	155	AY216594	104.3	0.996	0.993
*trx* ^6^	F:GGATTCCTTCTTCAGTGCCC R:GATGTCACAGTGTTTGGCCA	196	BT049355	98.6	0.999	0.998
*β-act* ^7^	F:CCAAAGCCAACAGGGAGAA R:AGGGACAACACTGCCTGGAT	102	AF012125	95.2	0.995	0.992

^1^ *hsp70*: Heat shock protein 70. ^2^ *hsp90β*: Heat shock protein 90B. ^3^ *igfbp1a*: Insulin-like growth factor binding protein 1A. ^4^ *igfbp1b*: Insulin-like growth factor binding protein 1B. ^5^ *ifna1*: Interferon alpha 1. ^6^ *trx*: Thioredoxin. ^7^ *β-act*: β-actin.

**Table 3:** Water quality parameters (mean ± standard error, SEM) measured during the experimental period ^1^.

Parameters	Water Quality During Trial			
	10 °C	14 °C	18 °C	22 °C
Temperature ^2^ (°C)	10.31 ± 0.17 ^a^	14.38 ± 0.11 ^b^	18.31 ± 0.08 ^c^	22.23 ± 0.09 ^d^
DO ^2^ (mg/L)	8.50 ± 0.07 ^ns^	8.43 ± 0.08	8.33 ± 0.18	8.31 ± 0.09
pH ^3^	7.79 ± 0.02 ^ns^	7.76 ± 0.05	7.78 ± 0.03	7.86 ± 0.02
Ammonia (mg/L)	0.00 ± 0.00 ^ns^	0.00 ± 0.00	0.00 ± 0.00	0.00 ± 0.00
Nitrite (mg/L)	0.16 ± 0.02 ^ns^	0.24 ± 0.01	0.19 ± 0.07	0.16 ± 0.06
Nitrate (mg/L)	2.91 ± 0.22 ^ns^	2.69 ± 0.18	2.93 ± 0.26	3.16 ± 0.23
SS (mg/L)	1.63 ± 0.35 ^ns^	1.77 ± 0.38	1.67 ± 0.41	1.32 ± 0.27

^1^ Values in each row with different superscripts are significantly different (*p* < 0.05). ns, not significant (*p* > 0.05). ^2^ Measured using a YSI DO meter (HQ40d). ^3^ Measured using a YSI pH meter (Pro1020).

**Table 4:** Growth performance of Atlantic salmon (*Salmo salar*) parr reared at different water temperatures for 60 days ^1^.

	Temperatures			
	10 °C	14 °C	18 °C	22 °C
Initial mean weight (g/fish)	31.78 ± 0.07 ^ns^	30.98 ± 0.38	31.19 ± 0.24	31.15 ± 0.17
Final mean weight (g/fish)	74.69 ± 1.31 ^b^	97.68 ± 1.26 ^a^	84.18 ± 6.92 ^b^	60.91 ± 1.53 ^c^
WG (%) ^2^	134.99 ± 4.20 ^c^	215.37 ± 3.88 ^a^	169.60 ± 5.78 ^b^	95.57 ± 5.85 ^d^
SGR (%/day) ^3^	1.42 ± 0.03 ^c^	1.91 ± 0.02 ^a^	1.65 ± 0.04 ^b^	1.12 ± 0.05 ^d^
DFI (%) ^4^	1.46 ± 0.02 ^b^	1.88 ± 0.02 ^a^	1.90 ± 0.10 ^a^	2.04 ± 0.03 ^a^
FE (%) ^5^	92.36 ± 2.83 ^a^	91.84 ± 1.53 ^a^	80.82 ± 2.11 ^b^	52.82 ± 2.98 ^c^
PER ^6^	1.88 ± 0.06 ^a^	1.87 ± 0.03 ^a^	1.65 ± 0.22 ^a^	1.08 ± 0.06 ^b^
CF ^7^	0.88 ± 0.01 ^ns^	0.89 ± 0.00	0.93 ± 0.01	1.02 ± 0.11
HSI (%) ^8^	1.33 ± 0.09 ^ns^	1.03 ± 0.09	1.33 ± 0.18	1.13 ± 0.09
VSI (%) ^9^	11.13 ± 0.26 ^a^	9.73 ± 0.73 ^ab^	9.90 ± 0.38 ^ab^	8.70 ± 0.42 ^b^
Survival (%) ^10^	95.38 ± 2.63 ^ns^	95.38 ± 2.63	96.83 ± 1.59	96.83 ± 1.59

^1^ Values are means from triplicate groups of Atlantic salmon. Different superscripts within a row indicate significant differences (*p* < 0.05). ns, not significant (*p* > 0.05). ^2^ Weight gain (WG, %) = (final weight (g) − initial weight (g))/initial weight (g) × 100. ^3^ Specific growth rate (SGR, %/day) = (ln final weight (g) − ln initial weight (g))/days × 100. ^4^ Daily feed intake (DFI, %) = feed intake/[(initial fish weight (g) + final fish weight (g) + dead fish weight (g)) × days reared/2] × 100. ^5^ Feed efficiency rates (FE, %) = (final weight (g) − initial weight (g))/feed consumed (g) × 100. ^6^ Protein efficiency ratio (PER) = wet weight gain (g)/protein intake (g). ^7^ Condition factor (CF) = [wet weight (g)/total length (cm)3] × 100. ^8^ Hepatosomatic index (HSI, %) = liver weight (g)/body weight (g) × 100. ^9^ Viscerasomatic index (VSI, %) = Viscera weight (g)/body weight (g) × 100. ^10^ Survival rate (%) = (final number of fish/initial number of fish) × 100.

**Table 5:** Whole-body proximate composition (% as-is) of Atlantic salmon (*Salmo salar*) parr at the end of the feeding trial ^1^.

	Temperatures			
	10 °C	14 °C	18 °C	22 °C
Moisture	69.73 ± 0.08 ^ab^	69.00 ± 0.13 ^c^	69.59 ± 0.16 ^b^	70.19 ± 0.15 ^a^
Crude protein	17.62 ± 0.03 ^ns^	18.81 ± 0.48	18.45 ± 0.86	18.15 ± 0.31
Crude lipid	10.07 ± 0.15 ^ns^	9.59 ± 0.59	9.09 ± 0.93	8.65 ± 0.40
Crude ash	2.38 ± 0.02 ^bc^	2.20 ± 0.01 ^c^	2.74 ± 0.09 ^a^	2.59 ± 0.05 ^ab^

^1^ Values are means from triplicate groups of Atlantic salmon. Different superscripts within a row indicate significant differences (*p* < 0.05). ns, not significant (*p* > 0.05). Initial whole-body proximate composition (mean ± SEM; n = 3) was as follows: moisture 70.31 ± 0.11%, crude protein 16.75 ± 0.29%, crude lipid 9.60 ± 0.33%, and crude ash 3.09 ± 0.09%.

**Table 6:** Plasma metabolites of Atlantic salmon (*Salmo salar*) parr reared at different water temperatures for 60 days ^1^.

	Temperatures			
	10 °C	14 °C	18 °C	22 °C
AST (U/l) ^2^	352.67 ± 31.06 ^ns^	319.00 ± 50.39	384.00 ± 108.45	358.00 ± 24.79
ALT (U/l) ^3^	7.33 ± 0.33 ^ns^	6.33 ± 0.33	7.33 ± 0.33	7.00 ± 0.58
GLU (mmol/dl) ^4^	5.44 ± 0.67 ^ns^	5.37 ± 0.58	6.04 ± 0.22	6.41 ± 0.16
TCHO (mmol/dl) ^5^	22.61 ± 0.89 ^ns^	20.71 ± 1.22	22.75 ± 1.70	23.78 ± 0.43
TP (g/dl) ^6^	6.00 ± 0.45 ^ab^	4.97 ± 0.32 ^b^	5.87 ± 0.28 ^ab^	6.80 ± 0.32 ^a^
TG (mmol/dl) ^7^	20.74 ± 0.35 ^ns^	17.61 ± 3.40	14.22 ± 1.48	20.31 ± 0.93
Na^+^ (mmol/l)	159.33 ± 17.13 ^ns^	159.33 ± 11.55	162.00 ± 1.53	167.33 ± 6.64
K^+^ (mmol/l)	1.37 ± 0.15 ^ns^	1.37 ± 0.09	1.27 ± 0.15	1.47 ± 0.15
Cl^−^ (mmol/l)	123.50 ± 3.67 ^ns^	142.67 ± 11.84	135.33 ± 4.33	142.67 ± 6.49
OSM (mmol/kg) ^8^	318.67 ± 4.18 ^ns^	314.00 ± 2.52	323.67 ± 3.38	330.00 ± 9.07

^1^ Values are means from triplicate groups of Atlantic salmon. Different superscripts within a row indicate significant differences (*p* < 0.05). ns, not significant (*p* > 0.05). ^2^ AST: Aspartate aminotransferase (=GOT). ^3^ ALT: Alanine aminotransferase (=GPT). ^4^ GLU: Glucose. ^5^ TCHO: Total cholesterol. ^6^ TP: Total protein. ^7^ TG: Triglycerides. ^8^ OSM: Osmolality.

**Table 7:** Stress and antioxidant responses of Atlantic salmon (*Salmo salar*) parr reared at different water temperatures for 60 days ^1^.

	Temperatures			
	10 °C	14 °C	18 °C	22 °C
Cortisol (ng/mL)	24.00 ± 2.44 ^ns^	21.83 ± 6.17	25.51 ± 2.53	17.16 ± 1.61
GPx (mU/mL) ^2^	57.08 ± 14.85 ^b^	679.33 ± 90.58 ^a^	124.95 ± 39.20 ^b^	371.47 ± 128.58 ^ab^

^1^ Values are means from triplicate groups of Atlantic salmon. Different superscripts within a row indicate significant differences (*p* < 0.05). ns, not significant (*p* > 0.05). ^2^ Glutathione peroxidase (mU/mL).

## Data Availability

The data supporting the findings of this study can be made available upon reasonable request by contacting the authors. The data are not publicly available due to privacy concerns.
